# Induction of Terminal Oxidases of Electron Transport Chain in Broccoli Heads under Controlled Atmosphere Storage

**DOI:** 10.3390/foods9040380

**Published:** 2020-03-25

**Authors:** Yoshio Makino, Jun Inoue, Hsiao-Wen Wang, Masatoshi Yoshimura, Kensaku Maejima, Sachiko Funayama-Noguchi, Takeshi Yamada, Ko Noguchi

**Affiliations:** 1 Graduate School of Agricultural and Life Sciences, The University of Tokyo, 1-1-1, Yayoi, Bunkyo-ku, 113-8657 Tokyo, Japan; jun19920701@gmail.com (J.I.); fyolxf@hotmail.com (H.-W.W.); ayoshimura@mail.ecc.u-tokyo.ac.jp (M.Y.); amaejima@mail.ecc.u-tokyo.ac.jp (K.M.); 2 Graduate School of Science, The University of Tokyo, 7-3-1, Hongo, Bunkyo-ku, 113-0033 Tokyo, Japan; funayama@bs.s.u-tokyo.ac.jp; 3 P-Plus Project, Sumitomo Bakelite Co., Ltd., 5-8, 2-chome, Higashi-Shinagawa, Shinagawa-ku, 140-0002 Tokyo, Japan; yamadada@sumibe.co.jp; 4 School of Life Sciences, Tokyo University of Pharmacy and Life Sciences, 1432-1, Horinouchi, Hachioji, 192-0392 Tokyo, Japan; knoguchi@toyaku.ac.jp

**Keywords:** alternative oxidase, *Brassica oleracea* var. *italica*, cytochrome *c* oxidase, mass loss, oxygen isotope discrimination

## Abstract

Controlled atmosphere (CA) storage, that is, at low O_2_ and high CO_2_ concentrations, effectively extends the shelf life of horticultural products. The influence of CA storage (O_2_/CO_2_: 2.5%/6.0% or 2.5%/0.0%) and in normal air (both at 1 °C for 21 d) on the physicochemical (O_2_ uptake, mass loss and L-ascorbate) and biological properties of broccoli (*Brassica oleracea* var. *italica*, Plenck, 1794) via amounts and activities of terminal oxidases of the electron transport chain was investigated. Mass loss, a sensitive index of freshness for broccoli heads under CA, was significantly lower under CA than under normoxia (*p* < 0.05). Mass loss was depressed 7 d earlier under CA, including 6.0% CO_2_ than under CA without CO_2_. High CO_2_ effectively depressed the degradation of L-ascorbate. During storage, the activity of the alternative oxidase (AOX) was lower under CA than in normal air (*p* < 0.05), while the amount of cytochrome *c* oxidase (COX), and the AOX/COX activity ratio (based on oxygen isotope discrimination), were not affected during storage. Our results indicate that CA storage effectively retained the freshness of broccoli heads by depressing the induction of AOX. However, depression of AOX amount was not associated with CO_2_ around broccoli heads.

## 1. Introduction

Refrigerated storage under controlled atmosphere (CA) conditions, that is, under reduced O_2_ and elevated CO_2_ concentrations, is a useful method to prolong the shelf life of many fruits and vegetables [1].

Broccoli (*Brassica oleracea* var. *italica*, Plenck, 1794) is known to be rich in micronutrients, such as vitamins, minerals, and flavonoids [2], and global production of this vegetable (reported as a sum of cauliflower and broccoli) increases annually (7.8-fold over the past 40 years) [3]. However, the quality of broccoli rapidly deteriorates after harvesting due to its high respiration activity [4]. Siddiqui et al. [5] reported loss of organoleptic quality and health-promoting compounds of fresh-cut broccoli florets. Li et al. [6] also reported head rot of broccoli caused by bacterial growth. Respiration is a main cause of mass loss [7] and degradation of nutrients [8] after harvest. CA storage at 0 °C with 2–5% O_2_ and 10% CO_2_ was reported to prolong the shelf life of broccoli heads by 1.5-fold compared to that under normal air [9]. Lipton and Harris [10] reported that shear resistance of broccoli heads was significantly retained by storing under 1% O_2_ and 10% CO_2_ at 5 °C or 7.5 °C for 3 d. Deschene et al. [11] reported that storage at 5 °C or 10 °C in a CA (O_2_/CO_2_: 3.0%/5.0%) strongly inhibited the loss of chlorophyll of cut heads of broccoli. Makhlouf et al. [12], studying CO_2_ production rate, color, chlorophyll concentration, soft rot, and mold reported that an atmosphere of 2.5% O_2_ and 6% CO_2_ is suitable for retaining freshness in broccoli heads.

CA storage extends shelf life by reducing metabolic activity, including respiration [4]. During respiration, stored nutrients are transformed to substrates that drive electron flow through the electron transport chain (ETC) [8]. This suggests that the activities of terminal oxidases of ETC are associated with the shelf life of horticultural products.

Wang et al. [13] reported that the induction of the alternative oxidase (AOX) in cut broccoli florets was depressed under atmospheres with low O_2_ and high CO_2_. AOX, the bypass of the cytochrome *c* oxidase (COX) pathway, is a nuclear-encoded protein located in the inner mitochondrial membrane, forming the alternative pathway that consumes O_2_, uncoupled from adenosine-5ʹ-triphosphate (ATP) production [14].

Depression of the AOX induction under atmospheres including low O_2_ and high CO_2_ was found by Wang et al. [13]. This phenomenon may also have been occurring in previous research [9,10,11,12]. However, in the study of Wang et al. [13], the storage temperature was 25 °C and they examined only the early stage of storage (within 50.5 h) conditions that differ from those used in practice for storage and transportation of broccoli heads. Furthermore, it was unclear whether the depression of AOX induction was caused by low O_2_ or high CO_2_ yet.

The objective of this study was to analyze the activities and amounts of two terminal oxidases (COX and AOX) of ETC under the CA conditions suggested by Makhlouf et al. [12] as suitable for storage of broccoli heads. Influence of low O_2_ or CO_2_ on freshness and terminal oxidase induction will also be separately investigated, differently from the study by Wang et al. [13]. The presented results help clarify the reason why CA storage is effective for retaining quality of horticultural products such as broccoli.

## 2. Materials and Methods

### 2.1. Samples

Forty-two freshly harvested (in Hokkaido, Aichi prefectures, Japan) heads of “Pixel” and “Ohayo” broccoli (*Brassica oleracea* var. *italica* Plenck) were sealed (18 for each cultivar) in an oriented polypropylene-based, micro-perforated pouch (Sumitomo Bakelite Co., Ltd., Tokyo, Japan; O_2_ transmission rate 7.65 × 10^5^ mL m^−2^ d^−1^ MPa^−1^; surface area 0.175 m^2^; thickness 25 μm, 18 pouches for one cultivar). Six heads (three for each cultivar) were used for 0 d samples. Heads of these two cultivars were easily obtained during the duration of the experiments. The micro-perforation results in an atmosphere inside the pouch was similar to ambient, while allowing maintenance of a very high relative air humidity (RH) inside.

### 2.2. Controlled Atmosphere Storage Methods

**Figure 1:**
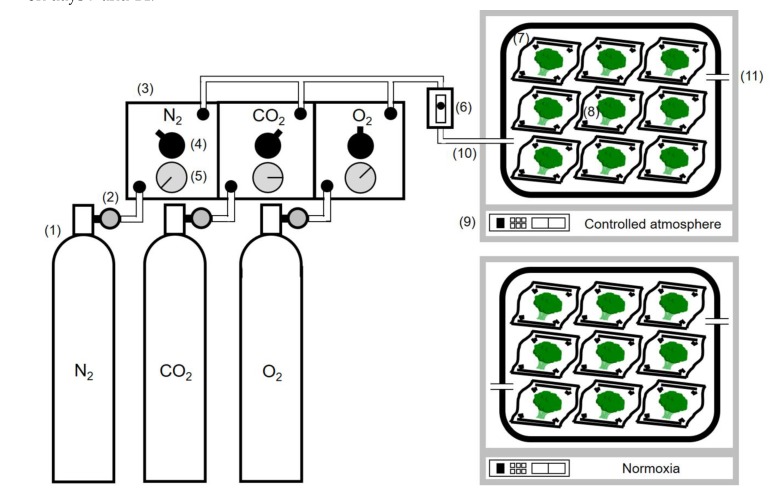
Schematic diagram of storage system for broccoli heads under controlled atmosphere and normoxia. (**1**) Gas cylinders, (**2**) regulators, (**3**) LogMIX gas mixture device (Fronto Co., Ltd., Kunitachi, Japan), (**4**) gas flow controller, (**5**) pressure gauge, (**6**) flowmeter, (**7**) 7-L acrylic jar (V-7, Shin-ei Sangyou Co., Ltd., Daito, Japan), (**8**) samples (broccoli heads), (**9**) temperature control units, (**10**) inlet, (**11**) outlet.

Storage experiments #1 (“Pixel”) and #2 (“Ohayo”) were conducted to investigate the effect of low O_2_/high CO_2_, or low O_2_, on freshness or terminal oxidases, respectively. One cultivar was used for one experiment. The storage system is shown in Figure 1. In experiment #1, nine heads sealed in micro-perforated pouches were enclosed in a 7 L acrylic chamber (V-7, Shin-ei Sangyou Co., Ltd., Daito, Japan) and exposed to a controlled atmosphere of 2.5% O_2_ and 6.0% CO_2_ (balanced with N_2_) at a constant flow rate of 100 mL min^−1^. Sealing in the pouch was conducted to prevent drying caused by air flow as associated with a mass loss of samples. In experiment #2, the gas composition was changed to O_2_ 2.5% and CO_2_ 0% (balanced with N_2_). In both experiments, another nine broccoli heads (controls) also sealed in micro-perforated pouches to retain the RH saturated point of the samples were stored under air without gas flow. The atmosphere in experiment #1 was the same atmosphere as reported by Makhlouf et al. [12]. Experiment #2 was conducted to investigate the influence of the removal of CO_2_ on the physicochemical and biological properties of the broccoli heads. In all experiments, the broccoli heads were stored at 1 °C for up to 21 d, and three of them were sampled on days 7 and 14.

### 2.3. O_2_ Uptake Rate Measurement

The O_2_ uptake of a broccoli head was measured at 1 °C, according to the method of Makino et al. [15]. A head was sealed in a laminated high-barrier pouch (AS ONE Co., Ltd., Osaka), which provided a closed system (O_2_ transmission rate, <9.87 mL m^−2^ d^−1^ MPa^−1^; surface area, 0.086 m^2^; thickness, 150 μm; polyethylene terephthalate/chlorinated polyethylene/aluminum/chlorinated polyethylene/polyethylene). O_2_ concentration in the headspace of the pouch was measured using a gas analyzer (CheckMate 3, Dansensor A/S, Ringsted, Denmark). The headspace volume was measured by the water displacement method. Equation (1) was used to calculate the O_2_ uptake rate, as follows:

(1) $$r=\frac{V\left(C_{0}-C_{t}\right)}{RTtm},$$

where:

*m* = mass of the broccoli head (kg)
*C* = O_2_ partial pressure in the pouch (MPa)
*t* = incubation time (h)
*T* = incubator temperature (K)
*r* = O_2_ uptake rate (mol kg^−1^ h^−1^)
*V* = void volume in the pouch (L)
*R* = universal gas constant (L MPa mol^−1^ K^−1^)
Subscript 0 = initial (start) time, subscript *t* = incubation time

### 2.4. Mass Loss Measurement

Mass loss, *m_L_* (%), was measured according to Equation (2) as:

(2) $$m_{L}=\frac{100\left(m_{0}-m_{t}\right)}{m_{0}}$$

### 2.5. L-Ascorbate Measurement

A 1 g sample of broccoli buds frozen in liquid N_2_ was homogenized with 5 g of 3% metaphosphate for 1 min and then centrifuged at 3000× *g* at 4 °C for 20 min using a MX-301 centrifuge (Tomy Seiko Co., Ltd., Tokyo, Japan). The supernatant was used for measurement of L-ascorbate. Sensor area of Ascorbic Acid Test (Merck KGaA, Darmstadt, Germany) was immersed in the supernatant and the degree of blue color of the sensor area was measured using a RQflex^®^ 10 reflective brightness meter (Merck KGaA). Then, the ascorbic acid concentration was observed in the display on the meter and expressed as the values on the wet basis.

### 2.6. Determinations of AOX and COX Protein Amounts

Amounts of AOX and COX protein produced by broccoli florets were measured to determine the relationship between floret O_2_ consumption and storage atmosphere. For immunoblots, tissue membranes were isolated according to Noguchi et al. [16]. Three grams of broccoli florets were crushed in a 20 mL grinding medium [0.3 mol L^−1^ sucrose, 25 mmol L^−1^ tetra-sodium pyrophosphate, 10 mmol L^−1^ monopotassium phosphate, 2 mmol L^−1^ ethylenediaminetetraacetic acid, 1 mmol L^−1^ glycine, 1% (*w*/*v*) polyvinylpyrrolidone-40, 1% (*w*/*v*) bovine serum albumin (BSA), 20 mmol L^−1^ ascorbate, and one tablet of a complete protease inhibitor (Roche, Mannheim, Germany) per 50 mL buffer (pH 7.5)]. The broccoli homogenate was centrifuged at 1100× *g* at 4 °C for 5 min. The supernatant was centrifuged again at 10,000× *g* at 4 °C for 5 min. The pellet was dissolved in 500 μL of sample buffer [62.5 mmol L^−1^ Tris-HCl (pH 6.8), 7.5% (*v*/*v*) glycerol, 2% (*w*/*v*) sodium dodecyl sulfate (SDS), 0.01% (*w*/*v*) bromophenol blue, and 50 mmol L^−1^ dithiothreitol]. Protein quantification was performed using the BSA as the standard. The preparation was diluted in 1 mL of sample buffer, denatured at 100 °C for 5 min, and separated by electrophoresis on 0.1% (*w*/*v*) SDS−12% (*w*/*v*) polyacrylamide gel according to Laemmli [17]. For the immunoreaction experiments, the proteins were transferred to a polyvinylidene difluoride membrane (Hybond-P, Amersham, Piscataway, NJ, USA). Anti-AOX (AS04 054, Agrisera, Vännäs, Sweden) and anti-COX II (AS04 053, Agrisera) were used as primary antibodies. Anti-Rabbit IgG, HRP-Linked Whole Ab Donkey (GE Healthcare, Fairfield, CT, USA) was used as the secondary antibody. The antibodies were diluted with a Can Get Signal (Toyobo Co. Ltd., Osaka, Japan). An enhanced chemiluminescence (ECL) Western Blotting Detection Reagent (GE Healthcare) was used as the chemiluminescent detection reagent and then detected with a CCD camera (LAS-4000, Fujifilm, Tokyo, Japan).

### 2.7. Determination of O_2_ Isotope Discrimination

Guy et al. [18] found that the discrimination factors of different O_2_ isotopes differed between AOX and COX. Therefore, the ratio of AOX to COX activity can be expressed by O_2_ isotope discrimination, hereafter abbreviated as “ *D*” (Equation (3)). The value of *D* for AOX is usually higher than that of COX [18]. Thus, to investigate the effect of various gas compositions on AOX and COX activities, the *D* value was measured according to the method of Wang et al. [13]:

(3) $$D=-\frac{\ln\left(\rho /\rho_{0}\right)}{\ln f},$$

where:

*D* = discrimination value (‰);
*ρ* = ^18^O/^16^O ratio of gas sample;
*ƒ* = fraction of in-package O_2_ concentration.

### 2.8. Statistics

All results were analyzed with Tukey’s honest significant difference test (level of significance was at 0.05) and two-way analysis of variance using JMP^®^ Pro ver.13.2.0 (SAS Institute, Cary, NC, USA).

## 3. Results and Discussion

### 3.1. Changes in O_2_ Uptake Rate

**Figure 2:**
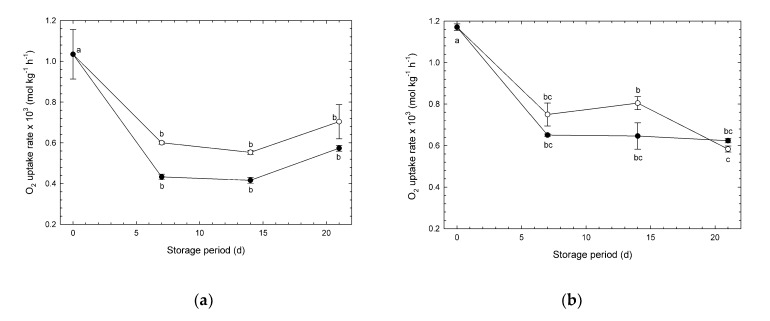
O_2_ uptake rates of broccoli heads during storage at 1 °C. (**a**) Experiment #1: ○, normoxia; ●, O_2_/CO_2_ = 2.5%/6.0% (+ N_2_ to 100%). (**b**) Experiment #2: ○, normoxia; ●, O_2_/CO_2_ = 2.5%/0.0% (+ N_2_ to 100%). Values are means ± SE of three biological replicates. Significant differences (*p* < 0.05) are denoted by different letters.

The changes in O_2_ uptake rate of broccoli heads under CA and normoxia are shown in Figure 2. In experiments #1 and #2, initial values were significantly higher than those during storage, possibly because the initial temperature of the samples was higher than that during storage. Oxygen uptake rates of “Ohayo” heads were higher up to 14 d than those of “Pixel”. The mean O_2_ uptake rates in the low O_2_ environment in both experiments were lower than those under normoxia except on day 21 in experiment #2. On individual sampling days, there were no significant differences between mean O_2_ uptake rates measured in low O_2_ and normoxia. Values measured during storage at 1 °C in the present study and those reported by Makhlouf et al. [8] were in the range of 0.59–0.80 mmol kg^−1^ h^−1^ and 0.26–0.63 mmol kg^−1^ h^−1^, respectively. These are much lower than the values reported elsewhere: 2.5–11 mmol kg^−1^ h^−1^ at 25 °C by Wang et al. [13]; 7.56 mmol kg^−1^ h^−1^ at 20 °C by Makino et al. [19]; and 9.99 mmol kg^−1^ h^−1^ at 20 °C by Robinson et al. [4]. The low values exhibited in the present study were likely caused by the low storage temperature, which also made it difficult to detect significant influences arising from the different atmospheres.

On the basis of two-way ANOVA, both the storage atmosphere and storage period significantly affected the O_2_ uptake rate in experiment #1 (Table S1). However, only the storage period significantly affected the O_2_ uptake rate in experiment #2 (Table S2). In experiment #1, the high concentration of CO_2_ was included in the atmosphere. Therefore, CO_2_ was effective in depressing the O_2_ uptake. The type of respiration of broccoli heads is reported as being in gradual decline [20]. Therefore, the O_2_ uptake rate significantly decreased over time in both experiments #1 and #2.

### 3.2. Mass Loss

**Figure 3:**
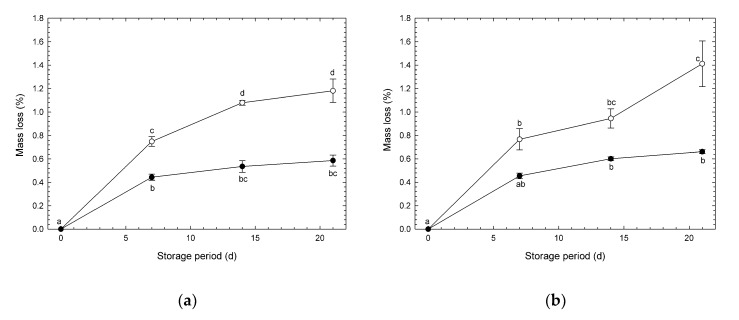
Mass loss of broccoli heads during storage at 1 °C. (**a**) Experiment #1: ○, normoxia; ●, O_2_/CO_2_ = 2.5%/6.0% (+ N_2_ to 100%). (**b**) Experiment #2: ○, normoxia; ●, O_2_/CO_2_ = 2.5%/0.0% (+ N_2_ to 100%). Values are means ± SE of three biological replicates. Significant differences (*p* < 0.05) are denoted by different letters.

Changes in mass loss of broccoli heads under CA and normoxia are shown in Figure 3. All the heads were sealed in micro-perforated pouches. Therefore, RH around the heads was maintained close to saturation. Also, gas flow into the CA chamber did not affect mass loss because the pouch effectively avoided influence by gas flow. Accordingly, mass loss was affected only by the environmental atmospheres around the samples. Mass loss under CA in experiment #1 was significantly lower by 0.30–0.60% than that under normoxia during days 7–21 from the start of storage. In contrast, mass loss under CA in experiment #2 was significantly lower by 0.75% than that under normoxia only on day 21. These results indicate that high CO_2_ combined with low O_2_ is effective in reducing mass loss in broccoli heads. Makhlouf et al. [12] reported that mass loss in broccoli heads was reduced under CA (O_2_/CO_2_: 2.5%/6.0%) at 1 °C after six weeks of storage. The decline in mass (i.e., mostly water) of broccoli heads reduces nutritional quality, salability (due to wilting, shriveling, softening, increased flaccidity, limpness, loss of crispness, and juiciness), and economic income, due to the loss of salable mass [21]. Mass loss of fruits and vegetables after harvest is mostly due to transpiration [7]. According to the results from Tables S1 and S2, CA storage appears to be effective in relating freshness (mass) of broccoli heads due to depression of transpiration. According to the results from Tables S1 and S2, mass loss was significantly reduced by both storage atmosphere and period. A low O_2_ condition was reported to be effective for depressing respiration [4]. Mass loss is caused by transpiration, which is associated with respiration [22]. Therefore, depression of O_2_ uptake under a low O_2_ condition was effective for depressing deterioration as mass loss.

### 3.3. L-Ascorbate

**Figure 4:**
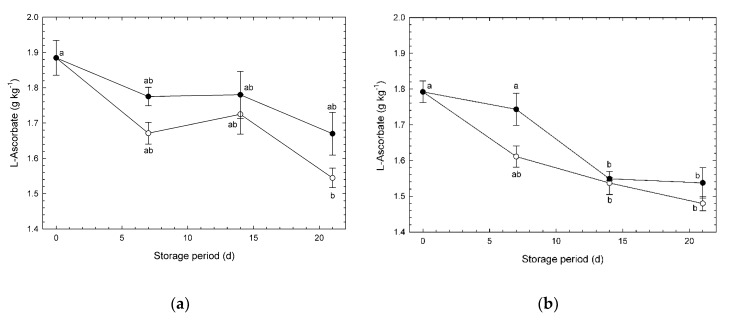
L-ascorbate concentrations in broccoli heads during storage at 1 °C. (**a**) Experiment #1: ○, normoxia; ●, O_2_/CO_2_ = 2.5%/6.0% (+ N_2_ to 100%). (**b**) Experiment #2: ○, normoxia; ●, O_2_/CO_2_ = 2.5%/0.0% (+ N_2_ to 100%). Values are means ± SE of three biological replicates. Significant differences (*p* < 0.05) are denoted by different letters.

Changes in L-ascorbate content in broccoli heads under CA and normoxia are shown in Figure 4. Initial concentration of L-ascorbate in “Pixel” broccoli heads was higher than those of “Ohayo”. This micronutrient performs various functions within plants, including roles as an antioxidant and as an enzyme co-factor, participation in photosynthesis, and involvement in cell wall metabolism [23]. This micronutrient is well-known as an indicator of freshness because its content decreases during storage [24,25]. Barth et al. [26] reported that L-ascorbate concentration in broccoli spears decreased over time at 10 °C, and modified atmosphere (O_2_/CO_2_: 10.0/8.0%) packaging was effective for retaining the concentration. In the present study, L-ascorbate concentration on day 21 under normoxia in experiment #1, and on and after day 14 both under CA and normoxia in experiment #2, were significantly lower than that in fresh heads. Only in heads stored under CA in experiment #1, did the L-ascorbate content not significantly decline during storage (Figure 4a), indicating that a high CO_2_/low O_2_ CA may be effective for retention of L-ascorbate content in broccoli heads. According to the previous report [25], degradation of L-ascorbate is responsible for catalysis by ascorbate peroxidase, a kind of oxidase. Oxygen uptake was significantly depressed under high CO_2_/low O_2_ CA (Table S1). Therefore, degradation of L-ascorbate may be significantly depressed under high CO_2_/low O_2_ CA.

### 3.4. Changes in Amounts of AOX and COX Enzymes during Storage

**Figure 5:**
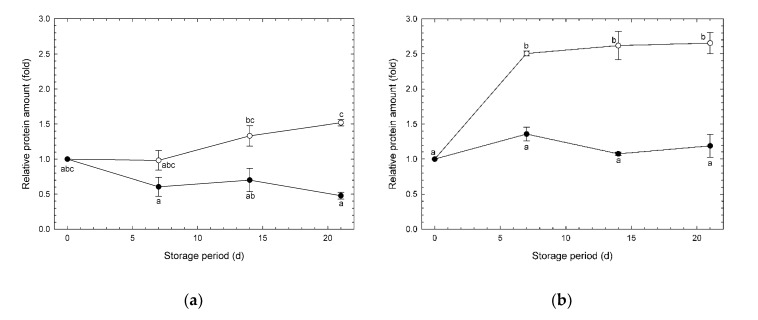
Relative amount of alternative oxidase (AOX) in broccoli heads during storage at 1 °C. (**a**) Experiment #1: ○, normoxia; ●, O_2_/CO_2_ = 2.5%/6.0% (+ N_2_ to 100%). (**b**) Experiment #2: ○, normoxia; ●, O_2_/CO_2_ = 2.5%/0.0% (+ N_2_ to 100%). Values are means ± SE of three biological replicates. Significant differences (*p* < 0.05) are denoted by different letters.

**Figure 6:**
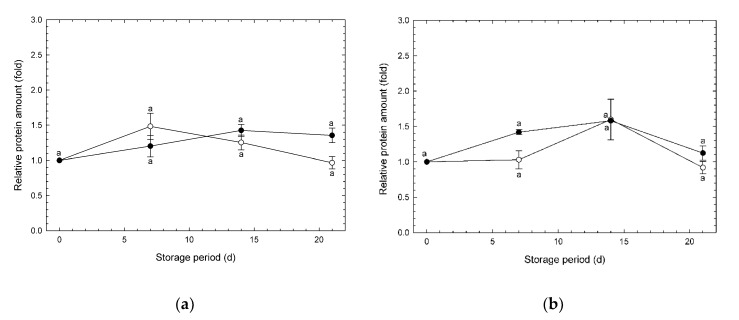
Relative amount of cytochrome *c* oxidase (COX) in broccoli heads during storage at 1 °C. (**a**) Experiment #1: ○, normoxia; ●, O_2_/CO_2_ = 2.5%/6.0% (+ N_2_ to 100%). (**b**) Experiment #2: ○, normoxia; ●, O_2_/CO_2_ = 2.5%/0.0% (+ N_2_ to 100%). Values are means ± SE of three biological replicates. Significant differences (*p* < 0.05) are denoted by different letters.

Changes in the amounts of AOX and COX enzymes in broccoli heads under CA and normoxia are shown in Figure 5 and Figure 6, respectively. In experiment #1, Amounts of AOX significantly declined during storage under CA but not under normoxia (Figure 5a, Table S1). In experiment #2, AOX amounts under CA were consistently smaller than those under normoxia (Figure 5b, Table S2). These results indicate that CA may inhibit the induction of AOX. AOX is an enzyme that consumes O_2_, and this small amount of AOX induction may be sufficient for the broccoli head under the low O_2_ environment. Wang et al. [13] reported that amounts of AOX in broccoli florets under a modified atmosphere (O_2_/CO_2_: 2.9–6.1%/10.0–11.0%) increased 2.83-fold, while those under normoxia increased 6.18-fold during 50.5 h storage at 25 °C. In contrast, Wang et al. [13] reported that AOX amounts under CA (O_2_/CO_2_: 6.0%/10.0%) at 32.5 h were almost the same as in fresh florets. This result from an early storage stage using cut samples is also reflected in the present research on long and cold storage using intact samples. According to the results of Table S1, O_2_ uptake rate and mass loss under CA was lower than those under normoxia. Depression of respiration is reported to contribute to depression of transpiration, a main cause of mass loss [22]. AOX is an oxidase that consumes O_2_ molecules. Therefore, depression of AOX induction may be effective for depressing mass loss as a serious deterioration phenomenon. According to the results from Table S2, mass loss and AOX induction were simultaneously depressed under CA, though the O_2_ uptake rate was not significantly depressed. The influence of environmental CO_2_ on the induction of AOX was not clear in the present study. Therefore, induction of AOX was found to be affected by only environmental O_2_ in the present study. This also suggested that AOX contributes to preventing over-reduction in biological tissue compared with COX, according to the results in Tables S1 and S2.

The amounts of COX were relatively stable during the storage period (Figure 6). According to the results from Tables S1 and S2, COX induction was significantly depressed under CA, including high CO_2_, though it was not depressed under CA without CO_2_. This suggests that high levels of CO_2_ affected induction of COX.

During respiration, stored nutrients, such as carbohydrates, lipids, and organic acids are transformed to substrates that drive H^+^ and e^−^ flows through the ETC. O_2_ molecules taken up by an entire plant are oxidized by AOX and COX. Therefore, oxidation by these enzymes promotes a reduction in stored nutrient concentrations, an effect that is one of the main causes of deterioration in horticultural products such as broccoli [8]. In the present study under CA, mass loss and induction of AOX were indices of deterioration in broccoli heads.

COX is the crucial terminal oxidase in oxidative phosphorylation, which directly reduces O_2_, and which is coupled with ATP synthesis [8,27]. ATP generated via oxidative phosphorylation is required to maintain the biological activity of plant cells [28]. These findings suggest that COX is important for maintaining metabolic activities in horticultural products after harvest. Therefore, the amount of COX may be maintained at a level suitable for maintaining metabolic activities.

In contrast, induction of AOX may be adjusted in relation to the environmental O_2_ concentration. There have been no reports of the measurement of terminal oxidases of ETC in horticultural products under long-term CA storage, despite the effectiveness of CA storage being recognized more than a century ago by Kidd [29,30,31]. When horticultural products are stored under normoxia, O_2_ more than CA is taken into cells, associated with induction of AOX or deterioration. Therefore, the effectiveness of CA for the retention of freshness may arise from the depression of induction of AOX. This hypothesis is suggested from the data for AOX and COX induction observed in the present study. However, whether this hypothesis could be applied universally needs further investigation using other kinds of horticultural products.

### 3.5. Changes in O_2_ Isotope Discrimination during Storage

**Figure 7:**
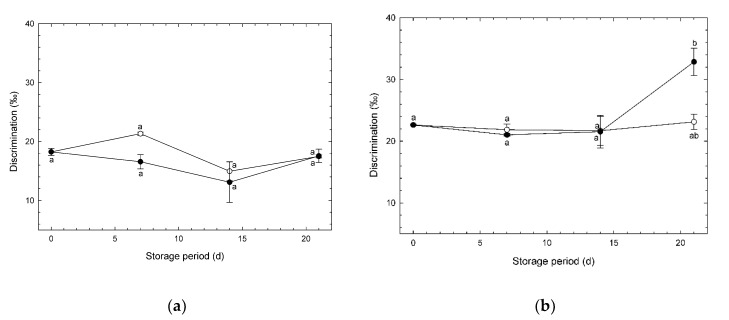
Discrimination factor (*D*) (O_2_ isotope discrimination factor for alternative oxidase/cytochrome *c* oxidase activity ratio) in broccoli heads during storage at 1 °C. (**a**) Experiment #1: ○, normoxia; ●, O_2_/CO_2_ = 2.5%/6.0% (+ N_2_ to 100%). (**b**) Experiment #2: ○, normoxia; ●, O_2_/CO_2_ = 2.5%/0.0% (+ N_2_ to 100%). Values are means ± SE of three biological replicates. Significant differences (*p* < 0.05) are denoted by different letters.

Changes in *D* of broccoli heads under CA and normoxia are shown in Figure 7. The initial value of *D* in “Ohayo” was higher than those in “Pixel”. *D* increases with an increase in AOX activity. Therefore, AOX activity may be affected specifically by cultivar. In experiment #1, *D* values were stable during storage, independent of atmosphere (Figure 7a). In experiment #2, the mean *D* value on day 21 under CA was significantly higher than that of fresh heads (day 0), and higher than that under normoxia on the same day (but not significantly). These results suggest that the influence of atmosphere on *D* values is minor, which reflects the results of Wang et al. [13]. According to Guy et al. [18], the *D* value is equivalent to the activity ratio of AOX to COX, and is higher with increasing AOX activity.

The influence of the atmosphere on the amount of AOX protein was clearly obvious in this study (Figure 5b), in contrast to the results for *D* (Figure 7). This suggests that the *D* value is less sensitive than AOX amounts for evaluating the effects of CAs on the biological properties in stored horticultural products. It may be difficult to evaluate the relationship between properties of terminal oxidases in broccoli heads and environmental atmospheres using *D*.

## 4. Conclusions

Amounts of AOX, but not COX protein seemed to depend on the storage atmosphere. The amount of AOX produced under hypoxia was significantly lower than that produced under normoxia, while the amount of COX produced was constant. Induction of terminal oxidases was independent of environmental CO_2_ in the present study. Product degradation, such as mass loss, which occurs during the storage of broccoli, was accompanied by an increase in the amount of AOX. Knowledge of the AOX-to-COX ratio under different storage environments may provide the basis for improving post-harvest storage strategies. The use of storage conditions that depress AOX induction may be useful for the reduction of post-harvest losses of horticultural products.

## Supplementary Materials

The following are available online at https://www.mdpi.com/2304-8158/9/4/380/s1, Table S1: Two-way analysis of variance on the effect of storage atmosphere and period on properties of broccoli heads stored for 21 d at 1 °C under two types of atmosphere (normoxia: O_2_/CO_2_ ≈ 21%/0.04%, CA (controlled atmosphere): O_2_/CO_2_ = 2.5%/6.0% (+ N_2_ to 100%)), Table S2: Two-way analysis of variance on the effect of storage atmosphere and period on properties of broccoli heads stored for 21 d at 1 °C under two types of atmosphere (normoxia: O_2_/CO_2_ ≈ 21%/0.04%, CA (controlled atmosphere): O_2_/CO_2_ = 2.5%/0.0% (+ N_2_ to 100%)).
