# Supplementary material for: Induction of Terminal Oxidases of Electron Transport Chain in Broccoli Heads under Controlled Atmosphere Storage
Source: Foods. 2020 Mar 25;9(4):380. doi: 10.3390/foods9040380 (PMC7230209; doi:10.3390/foods9040380)
Supplement: Supplementary file 1 [file foods-09-00380-s001.pdf]

Table S1. Two-way analysis of variance on the effect of storage atmosphere and period on properties of broccoli heads stored for 21 d at 1 °C under two types of atmosphere (normoxia: $O_2/CO_2 \approx 21\%/0.04\%$, CA (controlled atmosphere): $O_2/CO_2 = 2.5\%/6.0\%$ (+ N_2 to 100%)).

Variable	Effect	Degree of Freedom	Sum of Square	F	*p*
O_2 uptake rate (mol kg^{-1} h^{-1})	Storage atmosphere	1	9.48×10^{-5}	16.2	<0.0017 **
	Storage period	2	7.93×10^{-5}	6.78	<0.0107 *
	Interaction	2	1.19×10^{-6}	0.102	<0.9390
	Error	12	7.01×10^{-5}		
	Total	17	2.45×10^{-4}		
Mass loss (%)	Storage atmosphere	1	1.04	79.0	<0.0001 ***
	Storage period	2	0.267	10.1	<0.0027 **
	Interaction	2	0.0722	2.73	<0.1050
	Error	12	0.159		
	Total	17	1.54		
L-Ascorbate (g kg^{-1})	Storage atmosphere	1	4.04	3.96	<0.0698
	Storage period	2	7.08	3.46	<0.0649
	Interaction	2	0.387	0.189	0.8300
	Error	12	12.3		
	Total	17	23.8		
Alternative oxidase	Storage atmosphere	1	2.10	31.0	<0.0001 ***
	Storage period	2	0.184	1.36	0.2940
	Interaction	2	0.337	2.49	0.1250
	Error	12	0.813		
	Total	17	3.44		
Cytochrome *c* oxidase	Storage atmosphere	1	0.00318	0.117	0.7380
	Storage period	2	0.166	3.06	0.0844
	Interaction	2	0.488	9.02	0.0041*
	Error	12	0.325		
	Total	17	0.981		
Discrimination factor (‰)	Storage atmosphere	1	21.4	1.64	0.2240
	Storage period	2	77.0	2.96	0.0904
	Interaction	2	17.9	0.687	0.5220
	Error	12	156		
	Total	17	272		

* Significant at 95% level, ** Significant at 99% level, *** Significant at 99.9% level

Table S2. Two-way analysis of variance on the effect of storage atmosphere and period on properties of broccoli heads stored for 21 d at 1 °C under two types of atmosphere (normoxia: $O_2/CO_2 \approx 21\%/0.04\%$, CA (controlled atmosphere): $O_2/CO_2 = 2.5\%/0.0\%$ (+ N_2 to 100%)).

Variable	Effect	Degree of Freedom	Sum of Square	F	*p*
O_2 uptake rate (mol kg^{-1} h^{-1})	Storage atmosphere	1	2.36×10^{-5}	3.75	<0.0768
	Storage period	2	5.00×10^{-5}	3.94	<0.0483 *
	Interaction	2	3.14×10^{-6}	2.50	<0.1240
	Error	12	7.55×10^{-5}		
	Total	17	1.80×10^{-4}		
Mass loss (%)	Storage atmosphere	1	0.991	24.5	<0.0003 ***
	Storage period	2	0.555	6.86	<0.00103 **
	Interaction	2	0.179	2.22	<0.1520
	Error	12	0.486		
	Total	17	2.21		
L-Ascorbate (g kg^{-1})	Storage atmosphere	1	2.03	4.32	<0.0597
	Storage period	2	9.52	10.1	<0.0026 **
	Interaction	2	1.11	1.18	0.3400
	Error	12	5.63		
	Total	17	18.3		
Alternative oxidase	Storage atmosphere	1	1.59	13.0	<0.0036 **
	Storage period	2	0.215	0.876	0.4420
	Interaction	2	1.01	4.13	0.0431 *
	Error	12	1.47		
	Total	17	4.29		
Cytochrome *c* oxidase	Storage atmosphere	1	1.33	7.51	0.0179 *
	Storage period	2	1.01	2.84	0.0978
	Interaction	2	0.167	0.469	0.6360
	Error	12	2.13		
	Total	17	4.64		
Discrimination factor (‰)	Storage atmosphere	1	38.2	2.60	0.1330
	Storage period	2	167	5.66	0.0185 *
	Interaction	2	105	3.56	0.0612
	Error	12	177		
	Total	17	486		

* Significant at 95% level, ** Significant at 99% level, *** Significant at 99.9% level
